# Heart-targeting exosomes from human cardiosphere-derived cells improve the therapeutic effect on cardiac hypertrophy

**DOI:** 10.1186/s12951-022-01630-3

**Published:** 2022-10-04

**Authors:** Liang Mao, Yun-Da Li, Ruo-Lan Chen, Gang Li, Xiao-Xia Zhou, Fei Song, Chan Wu, Yu Hu, Yi-Xiang Hong, Xitong Dang, Gui-Rong Li, Yan Wang

**Affiliations:** 1 Xiamen Cardiovascular Hospital of Xiamen University, School of Medicine, Xiamen University, Xiamen, 361000 China; 2 Key Laboratory of Medical Electrophysiology, Ministry of Education & Medical Electrophysiological Key Laboratory of Sichuan Province, Collaborative Innovation Center for Prevention of Cardiovascular Diseases, Institute of Cardiovascular Research, Southwest Medical University, Luzhou, 646000 China; 3 Nanjing Amaigh Pharma Limited, Nanjing, 210032 China

**Keywords:** Genetic engineering, Exosomes, Heart homing peptide, Cardiosphere-derived cells, Cardiac hypertrophy

## Abstract

Exosomes of human cardiosphere-derived cells (CDCs) are very promising for treating cardiovascular disorders. However, the current challenge is inconvenient delivery methods of exosomes for clinical application. The present study aims to explore the potential to enhance the therapeutic effect of exosome (EXO) from human CDCs to myocardial hypertrophy. A heart homing peptide (HHP) was displayed on the surface of exosomes derived from CDCs that were forced to express the HHP fused on the N-terminus of the lysosomal-associated membrane protein 2b (LAMP2b). The cardiomyocyte-targeting capability of exosomes were analyzed and their therapeutic effects were evaluated in a mouse model of myocardial hypertrophy induced by transverse aorta constriction (TAC). The molecular mechanisms of the therapeutic effects were dissected in angiotensin II-induced neonatal rat cardiomyocyte (NRCMs) hypertrophy model using a combination of biochemistry, immunohistochemistry and molecular biology techniques. We found that HHP-exosomes (HHP-EXO) accumulated more in mouse hearts after intravenous delivery and in cultured NRCMs than control exosomes (CON-EXO). Cardiac function of TAC mice was significantly improved with intravenous HHP-EXO administration. Left ventricular hypertrophy was reduced more by HHP-EXO than CON-EXO via inhibition of β-MHC, BNP, GP130, p-STAT3, p-ERK1/2, and p-AKT. Similar results were obtained in angiotensin II-induced hypertrophy of NRCMs, in which the beneficial effects of HHP-EXO were abolished by miRNA-148a inhibition. Our results indicate that HHP-EXO preferentially target the heart and improve the therapeutic effect of CDCs-exosomes on cardiac hypertrophy. The beneficial therapeutic effect is most likely attributed to miRNA-148a-mediated suppression of GP130, which in turn inhibits STAT3/ERK1/2/AKT signaling pathway, leading to improved cardiac function and remodeling.

**Supplementary Information:**

The online version contains supplementary material available at 10.1186/s12951-022-01630-3.

## Background

Cardiac hypertrophy is initially a compensatory adaptive response to many cardiovascular diseases including chronic hypertension, aortic stenosis, mitral or aortic regurgitation, myocardial infarction, and genetic hypertrophic cardiomyopathy, among others [1]. Although current treatment regimens have significantly curtailed the progression of cardiac hypertrophy, unmet medical need remains in clinical practice. Therefore, novel therapeutics that can improve cardiac remodeling and heart functions are urgently needed.

Exosomes are nano-sized extracellular vesicles secreted from cells with ~ 30 to ~ 200 nm in diameter, and contain various signaling molecules that can be shuttled to recipient cells to modulate the pathophysiology of the latter [2]. Emerging evidence shows that exosome not only plays an important role in cell-cell communication in physiological processes, but also mediates the pathogenesis of many diseases and the therapeutic effect of cell therapy [3]. Cardiosphere-derived cells (CDCs) have been shown to reduce cardiomyocyte death, stimulate angiogenesis, suppress interstitial fibrosis, inhibit inflammation, and promote tissue regeneration after acute myocardial infarction [4, 5]. These therapeutic effects could be fully recapitulated by exosomes-derived from the CDCs, and abrogated by the inhibition of exosome secretion [6]. Moreover, retro-orbital injection of exosomes derived from human CDCs improved cardiac hypertrophy and dysfunction in an angiotensin II (Ang II)-induced cardiac hypertrophy mouse model [7]. Intra-myocardial injection of CDCs-exosomes in porcine acute myocardial infarction models significantly decreased cardiac remodeling and improved cardiac functions [8]. Although the therapeutic effect of CDCs-exosomes is very promising in the preclinical animal models, the translational potential is restricted. On the one hand, naturally occurring exosomes either lack or possess limited, tissue tropism, which tends to be trapped and quickly cleared by macrophages of the mononuclear phagocyte system in the liver, kidney, and lungs upon systemic delivery [9, 10]; and on the other hand, the procedures of retro-orbital injection, and open chest surgery followed by intra-myocardial or intracoronary artery injection require much more sophisticated skills and advanced cardiac monitoring facilities. Many studies have been conducted to overcome the poor circulation kinetics of exosomes. One of the most commonly used strategies is the exosome surface manipulation, where a ligand or homing peptide is either chemically conjugated to or displayed through genetic engineering of the parental cells on, the surface of exosomes, conferring exosomes targeting capability. Upon systemic administration, it shortens the time period to reach the therapeutic concentration of drugs to target organ, and thus increases the therapeutic efficacy [11, 12].

Various ligand/homing peptides have been successfully displayed on the surface of exosomes through forced expression of ligand/homing peptides fused in-frame with a transmembrane protein in the parental cells [13, 14]. Heart homing peptide (HHP) containing five amino acids CRPPR was previously discovered by phage display, which has been shown to localize in the heart tissue of mice after intravenous injection administration through specific binding to Cysteine-rich protein 2 (CRIP2) [15]. Exosomes displaying cardiac homing peptides have been shown to improve their internalization into cultured cardiomyocytes in vitro*,* and to target ischemia/reperfusion injured myocardium in vivo, leading to enhanced angiogenesis, decreased apoptosis and fibrosis, and improved cardiac functions [16, 17]. To explore whether the therapeutic effect of CDCs-exosomes can be improved by pan-cardiomyocyte targeting, the HHP was successfully displayed on the surface of exosomes derived from human CDCs, and the resulting exosomes were characterized, their targeting capability was confirmed*,* and their therapeutic effects to myocardial hypertrophy were evaluated in vitro in an Ang II-induced neonatal rat cardiomyocytes (NRCMs) hypertrophy cell model and in vivo in the TAC mouse model. Our results showed that exosomes displaying the HHP preferentially target to cultured NRCMs and accumulate in the hearts of mice upon systemic delivery, which greatly enhance the therapeutic effect of exosomes derived from CDCs to myocardial hypertrophy.

## Results

### Heart homing peptide is displayed on the surface of exosomes

**Fig. 1:**
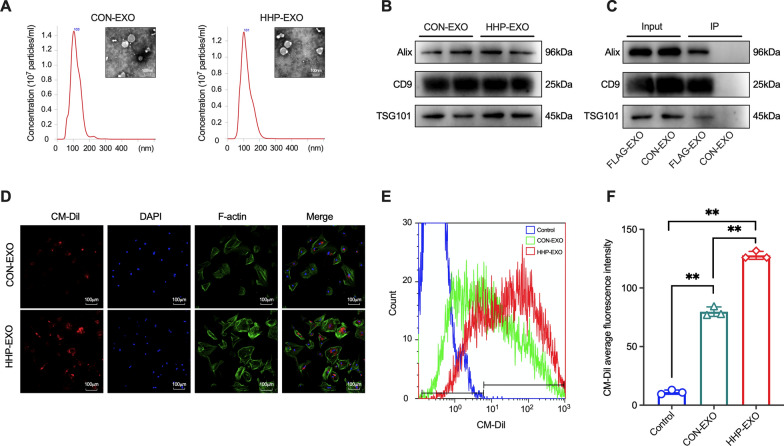
Characterization of exosomes and their internalization into NRCMs. **A** Representative images of CON-EXO and HHP-EXO under transmission electron microscopy (inlets) and their size distribution by NanoSight NS300. **B** Western blots showing the significant expression of the exosome markers CD9, TSG101 and Alix in CON-EXO and HHP-EXO. **C** Exosomes were immunoprecipitated with Anti-FLAG affinity resin and probed with antibodies against the exosome markers CD9, TSG101 and Alix. **D** Representative images showing the internalization of CM-Dil-labeled HHP-EXO or CON-EXO (red) into FITC-phalloidin stained NRCMs (green) and DAPI counter-stained nuclei (blue). **E** Typical flow cytometry plots showing the internalization of CM-Dil-labeled HHP-EXO or CON-EXO into NRCMs after 24 h incubation. **F** Mean fluorescence intensity in E (n = 3). Data are presented as ‘Mean ± SEM’, **P < 0.01

Exosomes can be manipulated to target specific tissues by surface display of a ligand or homing peptide [14]. To target cardiac tissue, plasmids encoding LAMP2b alone or HHP fused in-frame to LAMP2b were stably expressed in CDCs (Additional file 1: Figure S1), from which LAMP-exosomes (CON-EXO) and HHP-LAMP-exosomes (HHP-EXO) were prepared. Both CON-EXO and HHP-EXO were spherical or cup-shaped with an average diameter of 103 or 101 nm, respectively (Fig. 1A), and expressed the well-known exosome markers CD9, Alix, and TSG101 (Fig. 1B).

To demonstrate whether the homing peptide is displayed on the surface of exosomes, exosomes from CDCs expressing FLAG or LAMP were immunoprecipitated with anti-FLAG affinity resin, then immunoblotted with antibodies against exosome markers. As shown in Fig. 1C, immunoreactive bands with anti-CD9, anti-Alix and anti-TSG101 were apparently observed in FLAG-EXO, but not in CON-EXO, suggesting that FLAG is efficiently displayed on the surface of the exosomes, and thus inferring that HHP would be displayed on the surface of exosomes.

### HHP-EXO targets to cardiomyocytes in vitro and myocardium in vivo

To demonstrate the targeting capability, HHP-EXO or CON-EXO (10 μg) were labeled with Cell Tracker CM-Dil (in red), which were incubated with NRCMs for 24 h. The cells were fixed and stained with FITC-phalloidin (in green), and nuclei were counterstained with 4′6-diamidino-2-phenylindole (DAPI) (in blue). As shown in Fig. 1D, NRCMs incubated with HHP-EXO showed much stronger red fluorescence signal around nuclei than those incubated with CON-EXO. The enhanced internalization of HHP-EXO was further corroborated by flow cytometry analysis (Fig. 1E), in which the mean fluorescence intensity was greater in NRCMs incubated with HHP-EXO than CON-EXO (Fig. 1F), suggesting that more exosomes are accumulated in cardiomyocytes incubated with HHP-EXO than CON-EXO.

**Fig. 2:**
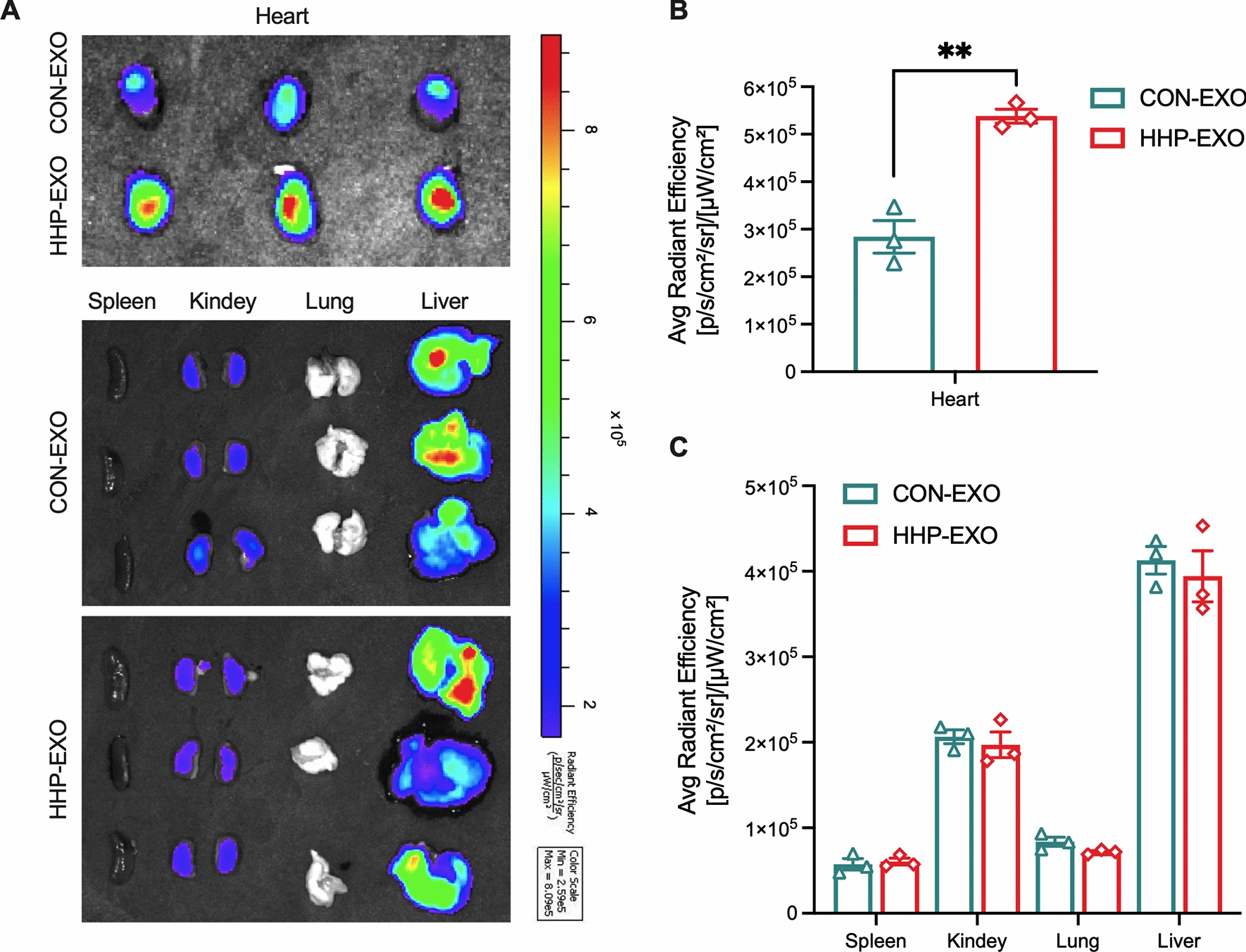
In vivo distribution of exosomes. CM-DiL-labeled CON-EXO or HHP-EXO was administered systemically for 24 h, and the hearts, spleens, kidneys, lungs and livers were harvested to evaluate their fluorescent intensity of the whole organs (**A**), and their mean fluorescent intensity in the hearts (**B**) and other organs (**C**) (n = 3). Data are presented as ‘Mean ± SEM’, with ** denoting P < 0.01

To evaluate the cardiac homing capability, exosomes labeled with CM-Dil were administered intravenously in mice (4 mg/kg) through tail-vein, and the animals were sacrificed post 24 h of the injection for collecting heart, liver, spleen, lungs, and kidneys to analyze the exosome retention using IVIS image system (IVIS, Caliper, USA) as described previously [18, 19]. The mean fluorescence intensity was almost doubled in hearts from mice receiving HHP-EXO compared to those receiving CON-EXO (Fig. 2A-B) (n = 3, P < 0.01). However, the fluorescence intensity was similar in liver, kidneys, lungs, and spleen in mice receiving HHP-EXO or CON-EXO although the fluorescence signal in liver was stronger than other organs (Fig. 2A-C). These results indicate that HHP-EXO possesses the capability to target cardiomyocytes in vitro and myocardium in vivo.

### HHP-EXO improves cardiac function in TAC mice.

**Fig. 3:**
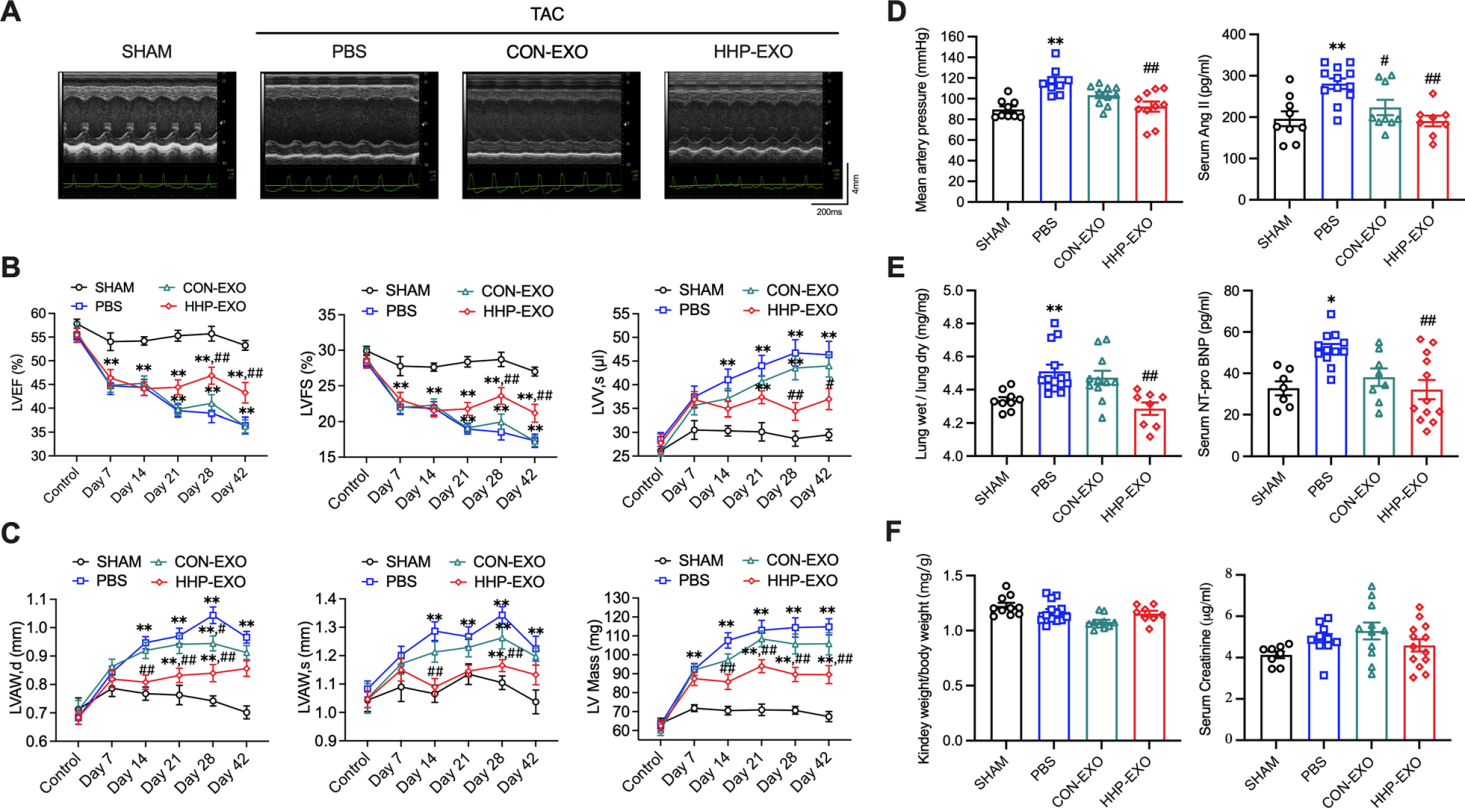
HHP-EXO improves cardiac function. **A** Representative 2D echocardiographic images in mice among groups. **B** Quantitation of LVEF, LVFS, and LVVs among groups (n = 8). **C** Quantitation of LVAWd, LVAWs, and left ventricular mass (n = 8). **D** Quantitation of mean artery blood pressure and serum Ang II level among groups (n = 8). **E** Quantitation of lung wet/dry weight and serum levels of NT-pro BNP among groups (n = 8). **F** Quantitation of the ratio of kidney/body weight and serum creatinine level (n = 8). Data are presented as ‘Mean ± SEM’, with *P < 0.05 and **P < 0.01(compared to sham); and ^#^P < 0.05 and.^##^P < 0.01 (compared to PBS)

The therapeutic potential of HHP-EXO to myocardial hypertrophy was studied in TAC mice with intravenous administration of CON-EXO or HHP-EXO (4 mg/kg) at day 8, 10, 12, 14, 16, 18, and 20 post-TAC (Additional file 1: Figure S2), and cardiac function was evaluated with echocardiography before and post-TAC (Fig. 3A). Compared to CON-EXO, HHP-EXO significantly ameliorated the TAC-induced decrease of left ventricular ejection fraction (LVEF) and left ventricular fractional shortening (LVFS) (Fig. 3B) at day 28 and day 42 post-TAC, respectively (P < 0.01 vs PBS). Compared to PBS treatment, left ventricular end-systolic volume (LVV.s) (Fig. 3B), diastolic left ventricular anterior wall thickness (LVAW.d), and systolic left ventricular anterior wall thickness (LVAW.s) (Fig. 3C) were slightly improved in the TAC mice treated with CON-EXO, but significantly improved in the TAC mice treated with HHP-EXO (P < 0.05 vs. PBS). Similarly, left ventricular mass (Fig. 3C) was slightly decreased in mice treated with CON-EXO, but significantly decreased in mice treated with HHP-EXO (Additional file 1: Table S1, P < 0.05 and P < 0.01 vs PBS, respectively). These results indicate that the targeting exosomes to the heart significantly improves the therapeutic effect of CDCs-exosomes to myocardial hypertrophy.

Left ventricular hypertrophy (LVH) is considered a biomarker for hypertension-induced organ damage [20]. The therapeutic effect of HHP-EXO to hypertension was evaluzted by analyzing mean blood pressure (MBP) of right carotid artery. Compared to sham animals, mice receiving PBS showed a significantly increased MBP (from 89.65 ± 3.00 to 116.70 ± 4.21 mmHg) (n = 9, P < 0.01) at 6 weeks post-TAC. The TAC-induced MBP increase was mildly improved (from 116.70 ± 4.21 to 103.40 ± 3.06 mmHg) (n = 10, P = 0.0963) in mice receiving CON-EXO, but significantly improved (from 116.70 ± 4.21 to 92.37 ± 4.91 mmHg) (n = 10, P < 0.01) in mice receiving HHP-EXO (Fig. 3D). These results indicate that HHP-EXO ameliorates TAC-induced blood pressure elevation.

Serum levels of Ang II and N-terminal pro B-type natriuretic peptide (NT-proBNP), markers of heart failure and remodeling, were significantly increased in TAC mice treated with PBS (Fig. 3D, E, n = 12, P < 0.01 vs. sham), which were reversed in TAC mice treated with CON-EXO or HHP-EXO (n = 8–12, P < 0.05 or P < 0.01 vs. PBS). The lung wet/dry weight ratio was increased in TAC mice receiving PBS, which was reversed in TAC mice by HHP-EXO (n = 8, P < 0.01), but not by CON-EXO (Fig. 3E). No significant changes were observed in the kidney/body weight ratio and serum creatinine level (Fig. 3F), indicating that the impairment of kidney function is limited in mice at 6 weeks post-TAC.

### HHP-EXO improves myocardial hypertrophy and fibrosis.

**Fig. 4:**
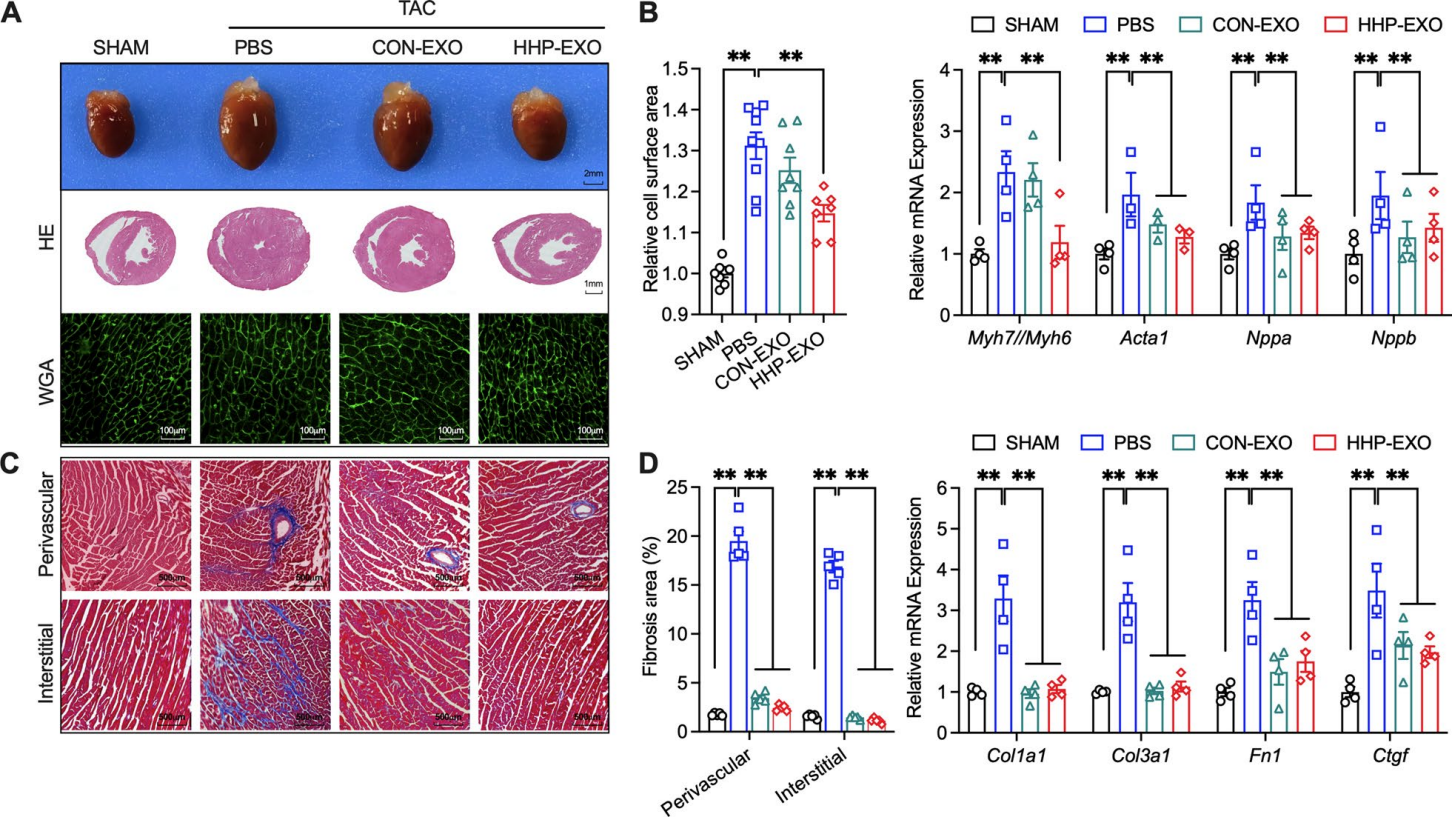
HHP-EXO improves TAC-induced myocardial hypertrophy and cardiac fibrosis. **A** Representative gross morphology of the hearts and the HE and WGA stained ventricular sections among groups. **B** Quantification of relative cell surface area of left ventricular sections stained with WGA in Fig. 4A (left panel). Quantification of the expression of *Myh7/Myh6* ratio, *Acta1*, *Nppa* and *Nppb* in left ventricular tissues of different animal groups as described in (A) (right panel) (n = 4–8). **C** Left ventricular sections stained with Masson’s staining (left panel) (n = 4–8). **D** Quantitation of fibrotic area in **C** (right panel) and the expression of fibrotic genes *Col1a1*, *Col3a1*, *Fn1* and *Ctgf* in left ventricular tissues among groups (n = 4–8). Gene expression was normalized to that of GAPDH. Data are presented as Mean ± SEM, with *P < 0.05 and **P < 0.01

The heart morphology, cross sections, and ventricular histology were analyzed in sham mice, TAC mice with PBS, CON-EXO and HHP-EXO (Fig. 4A–C). The heart size was clearly larger in TAC mice treated with PBS or CON-EXO than in sham mice, and smaller in TAC mice with HHP-EXO than in TAC mice with PBS or CON-EXO (Fig. 4A upper panel). The ventricular cross sections revealed that left ventricular wall thickness was greatly increased in TAC mice with PBS, which was slightly decreased in TAC mice with CON-EXO, but significantly decreased in TAC mice treated with HHP-EXO (Fig. 4A, middle panel). Consistently, the ratio of heart/body weight and the ratio of heart weight/tibial length were significantly increased in TAC mice treated with PBS relative to sham mice (n = 11, P < 0.01), this increase was significantly reversed in TAC mice receiving HHP-EXO (n = 9, P < 0.01), but not CON-EXO (Additional file 1: Figure S3).

Consistent with the results from histology analysis, the average cell surface area increased by 30% in TAC mice treated with PBS relative to sham mice (n = 9, P < 0.01), which was slightly decreased in TAC mice receiving CON-EXO (Fig. 4A lower panel, 4B left panel), but significantly decreased in TAC mice receiving HHP-EXO (n = 7, P < 0.01). Consistently, the expression of the hypertrophic marker genes *Acta1*, *Nppa*, and *Nppb* and the *Myh7/Myh6* ratio, were significantly increased (n = 4–6, P < 0.01) in TAC mice with PBS relative to sham mice (Fig. 4B, right panel). The increased expression of *Acta1*, *Nppa*, and *Nppb* was significantly ameliorated in TAC mice receiving both HHP-EXO or CON-EXO (n = 4–6, P < 0.01), whereas the *Myh7/Myh6* ratio was decreased only in TAC mice receiving HHP-EXO, but not CON-EXO (n = 6, P < 0.01).

The ventricular sections stained with Masson’s staining showed that perivascular and myocardial interstitial fibrosis of left ventricle were increased in TAC mice with PBS and decreased in TAC mice with CON-EXO or HHP-EXO (Fig. 4C). The perivascular fibrosis and myocardial interstitial fibrosis were significantly increased from 1.76 ± 0.07% and 1.57 ± 0.09% in sham mice to 19.49 ± 0.98% and 16.88 ± 0.59% in TAC mice treated with PBS (n = 5, P < 0.01), which were significantly decreased to 2.43 ± 0.14% and 1.13 ± 0.11% in TAC mice receiving HHP-EXO (n = 5, P < 0.01 vs PBS) or to 3.44 ± 0.26% and 1.48 ± 0.08% in TAC mice with CON-EXO, respectively) (n = 5, P < 0.01 vs PBS) (Fig. 4D, left panel). Consistently, the TAC-induced increase of fibrotic maker genes, *Col1a1*, *Col3a1*, *Fn1* and *Ctgf,* were remarkably reduced in TAC mice treated with HHP-EXO (n = 4–6, P < 0.01 vs. PBS) or CON-EXO (n = 4–6, P < 0.01 vs. PBS) (Fig. 4D, right panel). These results suggest that both HHP-EXO and CON-EXO improve TAC-induced myocardial fibrosis.

### HHP-EXO ameliorates TAC-induced increase of hypertrophy signal molecules.

**Fig. 5:**
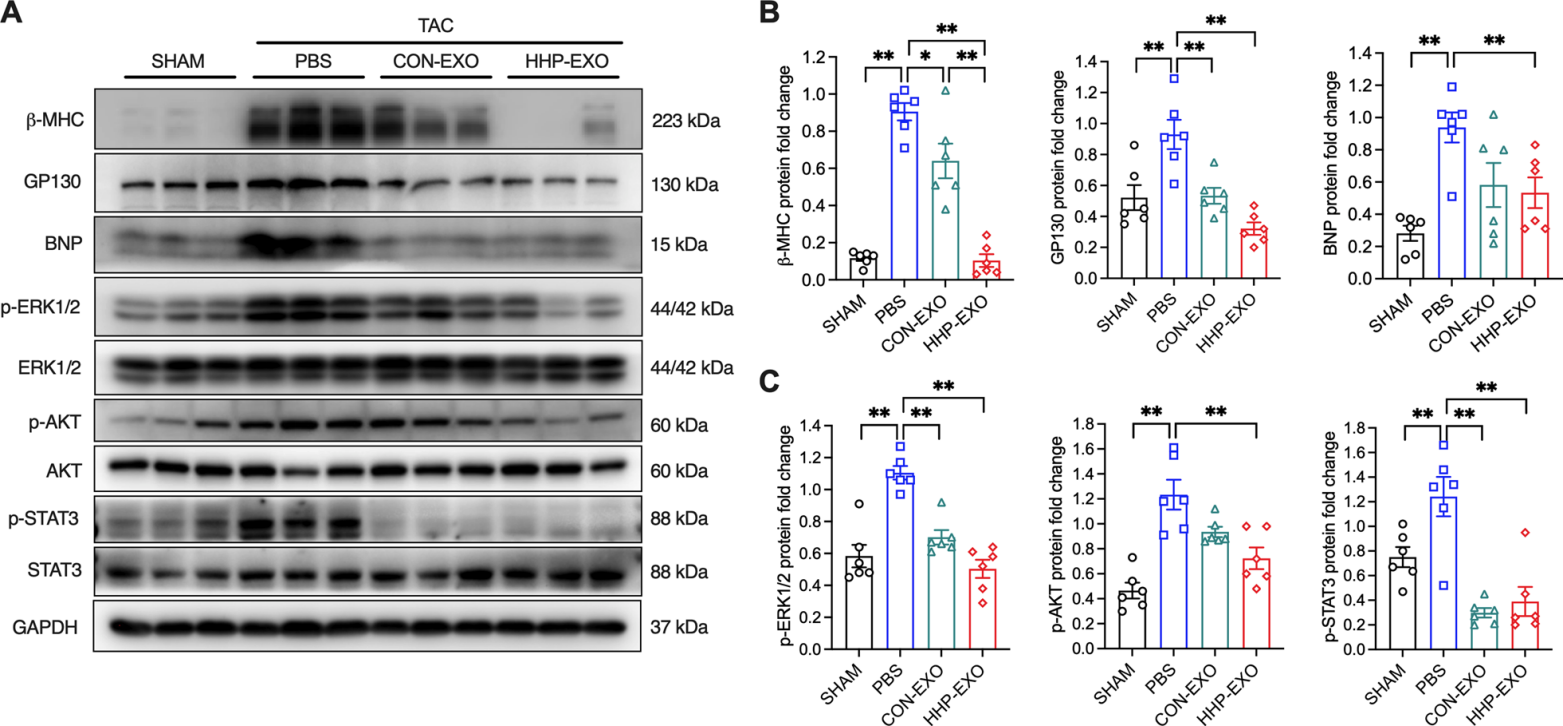
HHP-EXO inhibits TAC-induced expression of hypertrophy markers and the activation of ERK, AKT, STAT3 signaling pathways. **A** Representative images of Western blot showing the expression of β-MHC, BNP, GP130, STAT3, p-STAT3, ERK, p-ERK1/2, AKT and p-AKT in left ventricular tissues among different treatments. **B** Quantification of the expression of β-MHC, BNP and GP130 among groups (n = 4–7). **C** Quantification of the ratios of p-STAT3/STAT3, p-ERK1/2/ERK, and p-AKT/AKT among groups (n = 4–7). Protein expression was normalized to that of GAPDH. Data are presented as Mean ± SEM, with *P < 0.05 and **P < 0.01

To determine the molecular mechanism underpinning the beneficial effect of HHP-EXO, the cardiac hypertrophy-associated signaling molecules β-MHC, BNP, GP130, p-ERK1/2, p-AKT, and p-STAT3 [21] were analyzed in ventricular tissues of mice with different treatments. The protein expression of β-MHC, BNP, GP130, p-ERK1/2, p-AKT, and p-STAT3 was significantly up-regulated in TAC mice treated with PBS (n = 6, P < 0.01 vs sham mice), and the increased protein levels were significantly decreased in TAC mice receiving HHP-EXO (n = 6, P < 0.01 vs PBS), while only β-MHC, GP130, and p-STAT3 were decreased in TAC mice treated with CON-EXO (n = 6, P < 0.05) (Fig. 5). These results indicate that improvement of TAC hypertrophy by HHP-EXO is related to the inhibition of β-MHC, BNP, GP130, p-ERK1/2, p-AKT, and p-STAT3.

### miRNA-148a is enriched in CDCs-exosomes and successfully shuttled to the hearts upon systemic delivery

**Fig. 6:**
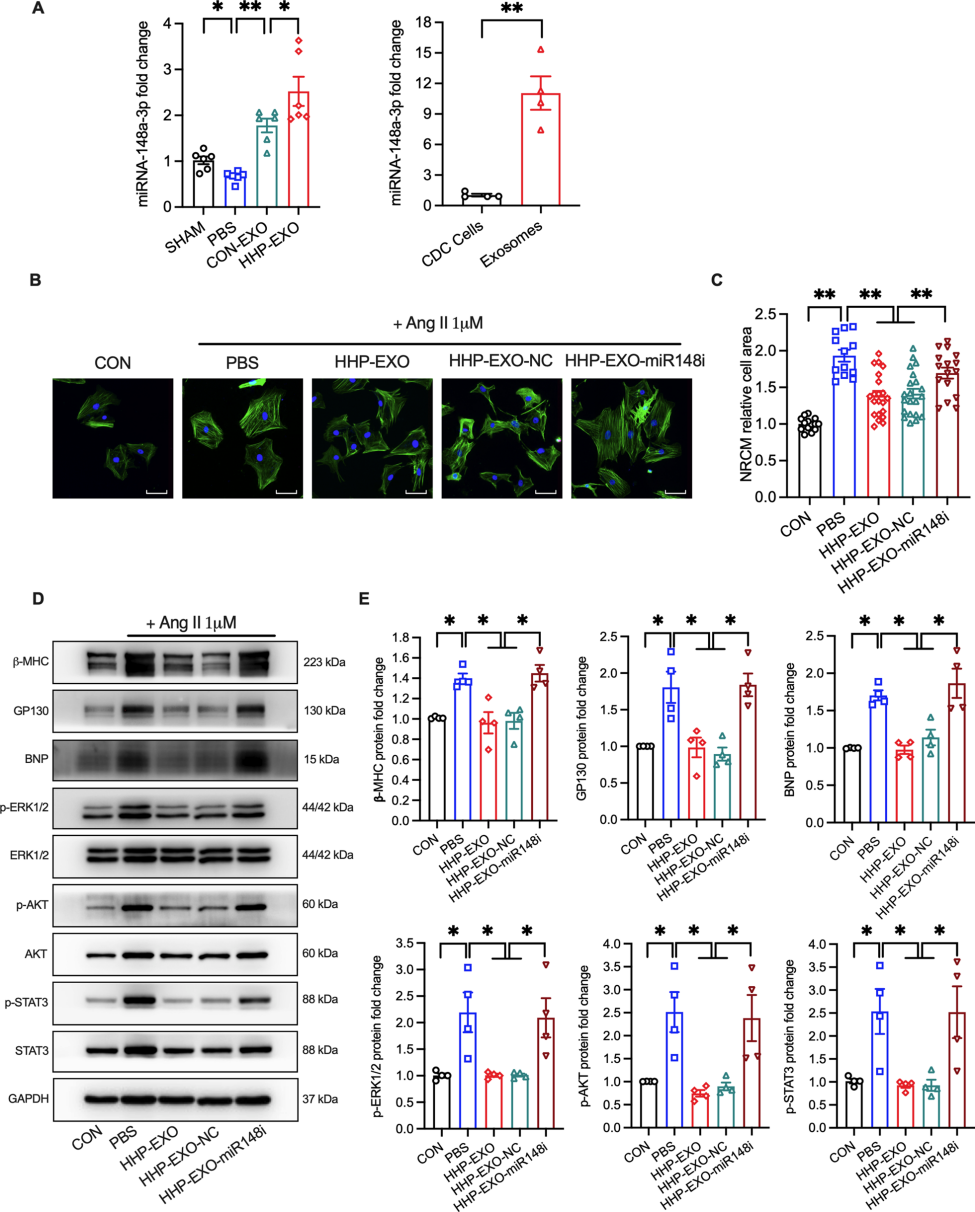
Exosomal miRNA-148a mediates the cardiac protective effect of HHP-EXO. **A** Relative levels of miRNA-148a in left ventricles among different treatment groups (left panel), and in CDCs and CDCs-derived exosomes (right panel). **B** Representative images of hypertrophic NRCMs induced by 1 µM Ang II and treated with PBS, HHP-EXO, HHP-EXO-NC or HHP-EXO-miRNA148i. NRCMs were stained with FITC-phalloidin and nuclei were counterstained with DAPI. **C** The mean cell surface area in **B** (n = 12–21 cells). **D** Representative images showing the expression of β-MHC, BNP, GP130, p-STAT3, STAT3, p-ERK1/2, ERK, p-AKT and AKT among groups in **B** (n = 4–6). **E** Quantitation of the expression of β-MHC, BNP, GP130, and ratios of p-STAT3/STAT3, p-ERK1/2/ERK, and p-AKT/AKT among groups in **B** (n = 4–6). The expression of miRNA-148a was normalized to that of U6, and protein expression was normalized to that of GAPDH. Data are presented as Mean ± SEM, with *P < 0.05 and **P < 0.01

It is generally believed that exosome-encased miRNAs play an important role in cell-to-cell communication. miRNA-148a is one of the most abundant encapsulated signaling molecules in CDCs-exosomes [22], which has been reported to protect the heart from pressure overload-induced systolic dysfunction via downregulation of GP130-STAT3 signaling pathway [23]. To investigate whether miRNA-148a is involved in the protective effect of HHP-EXO against TAC-induced cardiac hypertrophy, miRNA-148a level was determined in left ventricular tissues of mice with different treatments. miRNA-148a was signicantly reduced in the TAC mice treated with PBS (66.83% ± 4.78%) compared to that in sham group (101.83% ± 8.27%) (n = 6, P < 0.01) (Fig. 6A, left panel), which was reversed and increased to 177.80 ± 14.90% in TAC mice treated with CON-EXO (n = 6, P < 0.01) and to 252.30 ± 31.75% in mice treated with HHP-EXO (n = 6, P < 0.01). Coincidently, CDCs-derived exosomes selectively encapsulated miRNA-148a (> 11-fold) from parental CDCs (Fig. 6A, right panel). These results suggest that miRNA-148a most likely mediates the protective effect of HHP-EXO against cardiac hypertrophy.

### miRNA-148a mediates the protective effect of HHP-EXO against myocardial hypertrophy

**Fig. 7:**
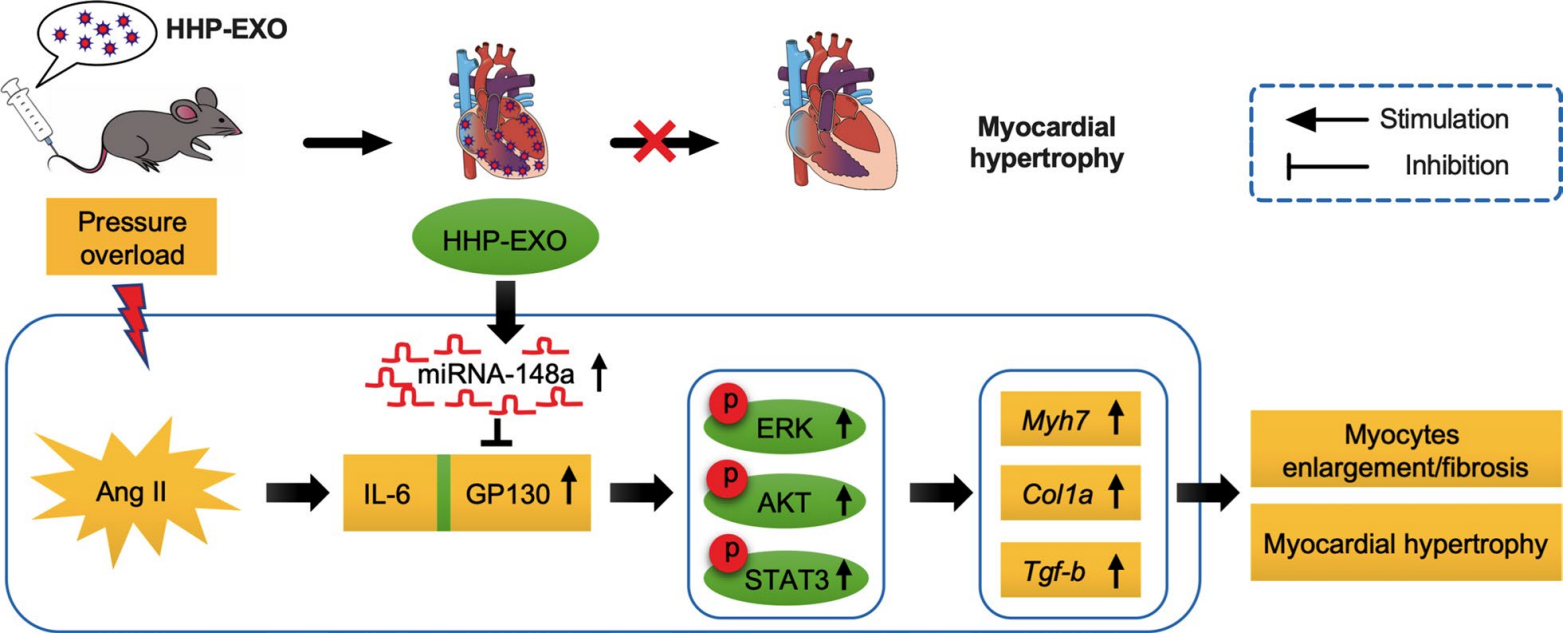
Schematic illustration of how exosomal miRNA-148a from HHP-EXO protects against pressure overload-induced cardiac hypertrophy

To examine the potential contribution of miRNA-148a to the protective effect of HHP-EXO against myocardial hypertrophy, miRNA-148a inhibitor (i) or scramble control (negative control, NC) was transfected into HHP-EXO, producing HHP-EXO-miR148i and HHP-EXO-NC respectively, and their effect was evaluated in Ang II-induced NRCMs hypertrophy in vitro. Ang II-induced increase of cell size (surface area) was significantly reduced by HHP-EXO or HHP-EXO-NC, but the effect was abrogated in HHP-EXO-miR148i treatment (Fig. 6B, C). Consistent with what observed in vivo, the protein expression of β-MHC, BNP, GP130, p-STAT3, p-ERK1/2, and p-AKT was significantly upregulated in Ang II-induced hypertrophic cardiomyocytes, and the effect was abolished in cells treated with HHP-EXO and HHP-EXO-NC, but not with HHP-EXO-miR148i (Fig. 6D, E). These results indicate that miRNA-148a mediates the protective effect of HHP-EXO against myocardial hypertrophy via down-regulation of GP130, leading to the inhibition of STAT3, ERK1/2, and AKT signaling pathways (Fig. 7). This is further supported by results with GP130 inhibitor SC144, in which SC144, like HHP-EXO, abolished Ang II-induced increase of β-MHC, GP130, p-STAT3, p-ERK1/2, and p-AKT in H9C2 cardiomyocytes (Additional file 1: Figure S4).

## Discussion

In this study, the HHP was successfully displayed on the surface of exosomes derived from human CDCs, which conferred the exosome capability to target NRCMs in vitro and were selectively enriched in the hearts after systemic delivery. The HHP-EXO ameliorated Ang II-induced cardiomyocyte hypertrophy in vitro and significantly improved cardiac remodeling and functions in the TAC-induced myocardial hypertrophy compared to its non-targeted counterpart. Mechanistically, miRNA-148a, that was selectively encapsulated into CDCs-exosomes mediated the therapeutic effect of HHP-EXO to myocardial hypertrophy via down-regulation of the expression of GP130, leading to inhibition of STAT3, ERK1/2 and AKT signaling pathways.

Exosomes that selectively enrich signaling molecules of parental cells can be shuttled to recipient cells through paracrine or hormone-like mechanisms, mediating many pathophysiological processes, such as angiogenesis, proliferation, and apoptosis and inflammation [24–26], among others. Increasing evidence shows that exosomes mediated the therapeutic effect of stem cell transplantation immediately after acute myocardial infarction (AMI) [27]. Human CDCs express stem cell markers and proteins critical for cardiomyocyte contraction and electrical function [28]. Similar to stem cell therapy, the early reports that intracoronary infusion of autologous CDCs in AMI patients and intramyocardial injection of human CDCs in porcine models after AMI significantly improved cardiac functions, which could be recapitulated by CDCs-derived exosomes [6, 29–31]. Given exosomes’ high stability, low immunogenicity and toxicity, biocompatibility, and selectively enriched certain signaling molecules, CDCs-exosomes could be explored for their therapeutic potential to treat cardiac diseases.

Naturally occurring exosomes generally display short half-life and possess very limited organotropism and capability to home to where the parental cells were originated [32]. After systemic administration, these native exosomes were mainly trapped in the liver, spleen, kidneys, and lungs, where they were cleared by macrophages of the mononuclear phagocyte system within 10 min in mice. In contrast, surface modified exosomes could be detected in the plasma of the mice 60 min after systemic injection [18, 33, 34]. Indeed, native CDCs-exosomes after systemic administration were localized mainly to the liver, spleen, and lungs in mice, whereas CDCs-exosomes displaying cardiac homing peptides showed much higher uptake by cardiomyocytes at 2 and 24 h, larger decrease of cardiomyocyte apoptosis, and higher cardiac retention, than their non-targeted counterparts after intramyocardial injection [17]. Consistently, we showed that CDCs-exosomes displaying the HHP neither alter the physical characteristics of native CDCs-exosomes in terms of morphology, size distribution, and surface markers (Fig. 1), and nor change the distribution profile of exosomes as both HHP- and CON-EXO were comparably trapped in the liver, spleen, lungs, and kidneys. These HHP-EXO were preferentially internalized into cultured cardiomyocytes, and were significantly retained in the heart at 24 h after systemic delivery (Fig. 2).

miRNA is one of the most commonly encapsulated signaling molecules in exosomes that modulate the biology of recipient cells [35]. CDCs-exosomes have been reported to selectively enrich miRNA-148a, miRNA-181, miRNA-146, and miRNA-210, which improved cardiac function in clinical trials and various animal models of AMI and other heart diseases [22, 24, 36, 37]. In a TAC mouse model, exogenous miRNA-148a inhibited the expression of glycoprotein 130 (GP130) which in turn suppressed the phosphorylation of STAT3 and the expression of cardiac remodeling related genes, leading to improved ventricular dilatation and heart failure [23]. Coincidently, we found that miRNA-148a was selectively enriched in CDCs-exosomes that, upon systemic delivery, significantly restored the TAC-induced down-regulation of miRNA-148a, and improved cardiac hypertrophy in the TAC model (Figs. 3, 4, 6).

GP130 is a constitutively expressed signaling molecule that forms a receptor complex relaying signals for several cytokines [38]. It plays a critical role in the normal development of cardiovascular systems and participates in the pathogenesis of cardiac hypertrophy through activation of JAK/STAT1/3, Ras/ERK1/2, and PI3K/AKT signaling pathways [39–41]. Inhibition of STAT3 activation by celastrol decreased cardiac fibrosis and hypertrophy in Ang II- and TAC-induced cardiac hypertrophy mouse models and in rat renal artery stenosis model [42, 43]. Furthermore, we showed that both CON-EXO and HHP-EXO suppressed the phosphorylation of STAT3, the expression of fibrosis-related genes, and deposition of myocardial collagen. Importantly, HHP-EXO significantly inhibited cardiac hypertrophy related genes, and suppressed the tissue levels of GP-130, p-STAT3, p-ERK1/2 and p-AKT. Importantly, inhibition of GP130 with SC144 in the presence of HHP-EXO showed no additional inhibitory effect of the decreased expression of hypertrophic marker proteins (Additional file 1: Figure S4). We speculate that the observed enhanced anti-cardiac hypertrophy effect of HHP-EXO is most likely mediated by exosomal miRNA-148a that, in turn, inhibitis the activation of GP130 followed by reducing pSTAT1/3, pERK1/2, and pAKT signaling pathways.

It is generally recognized that TAC-induced cardiac hypertrophy is associated with increase of artery blood pressure, which is involved in activation of renin-angiotensin system (RAS) [44, 45]. The present study also showed that mean artery blood pressure was significantly elevated in TAC mice cardiac hypertrophy with increase of serum Ang II. Interestingly, the mean blood pressure in TAC mice treated with intravenous HHP-EXO or CON-EXO was lower than TAC mice with PBS. The potential mechanism of this effect is likely related to inhibiting RAS, because serum Ang II levels were similarly decreased in TAC mice treated with HHP-EXO or CON-EXO.

The previous reports demonstrated that biodistribution of exosomes was relatively stable within 24 h after administration [18, 19]. We therefore performed the biodistribution assay post 24 h intravenous administration of exosomes. The limitation of the present study was lack of more informative biodistribution data at more time points to learn the effect of intravenously administered HHP-EXO on the heart tissues. However, it wouldn’t affect the conclusion that HHP-EXO enhanced the therapeutic effect of CDCs-derived exosomes against TAC-induced cardiac hypertrophy.

Collectively, the present study demonstrates that HHP-exosomes preferentially target the heart and enhance the therapeutic effect of CDCs-exosomes on cardiac hypertrophy. The beneficial therapeutic effect is most likely related to miRNA-148a-mediated inhibition of GP130 followed by supressing STAT3/ERK1/2/AKT signaling pathway, leading to improved cardiac function and remodeling.

## Methods

### Reagents

Reagents and antibodies used in this work are listed in Additional file 1: Table S2.

### Plasmid construction of heart homing peptide

The heart homing peptide (CRPPR, HHP) fused in-frame with human Lamp2 (NM_013995.2, hLAMP2b) between amino acid 31–32 was generated by overlapping polymerase chain reaction (PCR) using primers shown in Additional file 1: Table S3 [46]. The PCR products were purified and cloned into pLVX-IRES-ZsGreen1 plasmid with XhoI and BamHI restriction enzymes, generating plasmid pLVX-IRES-ZsGreen1-HHP-hLAMP2b (HHP-pLVX). Using the same strategy, a FLAG Tag (DYKDDDDK) was fused in-frame with LAMP2b, generating plasmid pLVX-IRES-ZsGreen1-FLAG-hLAMP2b (FLAG-pLVX). The plasmids were sequenced to confirm their identities (Qingke Biotech Inc., Chengdu, China).

### Preparation of human cardiosphere-derived cells and exosomes

Human atrial specimens were obtained from patients underwent heart surgery at Xiamen Cardiovascular Hospital, Xiamen University. Human cardiosphere-derived cells (CDCs) were isolated and cultured as described previously [47]. Briefly, atrial specimen was minced into small pieces of approximately 1–2 mm^3^, which were enzymatically digested and then plated to allow the cardiac explants to grow. After 5–7 days, cells surrounding the explants were harvested and seeded onto poly-D-lysine-coated dishes to allow cardiosphere formation. Two days later, cardiospheres were collected and plated on fibronectin-coated dishes to generate CDCs. The CDCs was expanded to at least passage 4 for downstream experiments.

CDCs of passage 4 were transfected with lentiviral hLAMP2b (LAMP), HHP and FLAG, respectively. The stable cell lines expressing LAMP (LAMP-CDCs), HHP (HHP-CDCs), and FLAG (FLAG-CDCs) were established by sorting green fluorescent protein (GFP) positive cells using flow cytometry. Stable cell lines, LAMP-CDCs and HHP-CDCs were expanded to passage 8 in Iscove's Modified Dulbecco's Media (IMDM) supplemented with 10% FBS. The cells were refreshed with serum-free IMDM and further incubated for 2 weeks. The conditioned medium (CdM) was harvested, filtrated through a 0.45 μm filter, and then stored at − 80 °C. Exosomes from HHP-CDCs and LAMP-CDCs were isolated from the CdM by gradient centrifugation as described previously [48]. Briefly, the CdM was thawed, then spun at 110,000 × g for 2 h at 4 °C. The pellet was dissolved into cold PBS, and spun again at 110,000 × g for 70 min at 4 °C. The pellet was then dissolved in cold PBS and stored at −80 °C for further analysis and applications.

### Preparation of neonatal rat cardiomyocytes and treatment with Angiotensin II

Neonatal rat cardiomyocytes (NRCMs) were isolated by collagenase and trypsin enzymatic dissociation from 1–2 days old newborn rat hearts as previously described [49]. NRCMs were seeded onto 6-well plates in Dulbecco’s Modified Eagle Medium (DMEM) supplemented with 20% fetal bovine serum (FBS) and penicillin/streptomycin. After 48 h grown at 37 °C with 5% CO_2_, NRCMs were subjected to 1 μM Ang II treatment in the presence of 50 μg/ml exosomes or controls for 48 h. H9C2 cardiomyocytes were seeded onto 6-well plates in DMEM supplemented with 10% FBS and penicillin/streptomycin. After 24 h grown at 37 °C with 5% CO_2_, H9C2 cells were pretreated with 10 μM SC144 for 1 h, and then exposed to 1 μM Ang II with or without 50 μg/ml HHP-EXO for 24 h.

### Transverse aortic constriction model

Transverse aortic constriction (TAC) surgery was performed on 10-week-old C57BL/6 male mice [50]. Briefly, mice were anesthetized, intubated, and placed on a ventilator. Sternotomy was performed to expose the aorta, and a 6–0 propene suture was placed around the aorta and tightened around a blunt 27-gauge needle positioned between the right innominate artery and left common carotid artery of the aortic arch. The needle was then removed, and the chest was closed. At day 7 after the TAC, cardiac function was evaluated with echocardiography to confirm that the TAC procedure was successful. Animals with unsuccessful ligation (no change in peak flow velocity of aortic arch on the site of the constriction) were excluded from the study. The mice with successful TAC were randomly divided into 3 groups (n = 12), PBS control, CON-EXO, and HHP-EXO groups. Starting on day 8 post-TAC, 4 mg/kg exosomes were tail-vein injected on every third day for a total of 7 times.

### Echocardiography and arterial blood pressure measurement

Cardiac function and gross morphology were assessed by echocardiography using Vevo 2100 (Visual Sonics, Canada) equipped with a 40-MHz MS550D probe and a high-frequency ultrasound system as described previously [49]. Echocardiograms were performed 3 days before the surgery (Control), and on day 7, 14, 21, 28 and 42 post-TAC. Arterial blood pressure was measured on day 42 using the carotid artery catheter method [51]. Briefly, mice were anesthetized by 2% isoflurane and body temperature was kept at 37 °C. The right carotid artery was isolated, clamped and intubated with a PE10 catheter pre-filled with heparin solution (0.1 IU/ml in saline). The catheter was connected to RM6240 multichannel physiological signal acquisition and processing system with a YPJ01 pressure transducer (Chengdu Instrument Factory, China). Aortic pressure was recorded for approximately 30–60 s.

### Histological analysis

Hearts, lungs and kidneys were harvested after blood pressure measurement, rinsed in saline, and weighed. Lungs were dried in an oven at 60 °C for 5 days and re-weighed as dry weight. Hearts were embedded in optimal cutting temperature (OCT) medium. Frozen sections of 6.0 μm were fixed in 4% paraformaldehyde, and stained with H&E (Hematoxylin-eosinstaining) and WGA (Wheat Germ Agglutnin) WGA and Masson’s trichrome solutions, respectively [49]. Slides were observed and images were acquired using the tissue cytometry system TissueFAXS (TissueGnostics, Austria). Cardiac fibrosis of the LV was evaluated using ImageJ software.

### Western blot

Western blot was performed as previously described [49]. Briefly, equal amount of protein lysates was resolved on 10% polyacrylamide gel and transferred to PVDF membranes. The membranes were incubated respectively with primary antibodies against GP130, phospho-p44/42 MAPK (ERK1/2) (Thr202/Tyr204), phospho-AKT (Ser473), p44/42 MAPK (ERK1/2), AKT, β-MHC, BNP, phospho-STAT3 (p-Tyr705), STAT3, and GAPDH (Additional file 1: Table S2), for overnight at 4 °C. The membranes were washed and incubated with their corresponding species-specific horseradish peroxidase (HRP) conjugated secondary antibodies. The immunoreactive bands were developed with enhanced chemiluminescence (ECL) and images were acquired and quantitated on BioRad ChemiDoc MP imaging system.

### Statistical analysis

Statistical analyses were performed using GraphPad Prism 9 software. Unpaired two-tailed Student’s t test was employed to determine the differences between two groups or one-way ANOVA followed by Tukey’s post hoc test was used for comparison among multiple groups. Date were presented as mean ± SEM, and P < 0.05 was considered statistically significant.

## Supplementary Information

**Additional file 1: Figure S1. **Schematic illustration of displaying a homing peptide/FLAG on the surface of exosomes. The coding sequence of HHP/FLAG (red bar) was fused in-frame to the LAMP2b cDNA between the signal peptide (SP) and the N-terminus, which was then cloned into pLVX-IRES-ZsGreen1 expression plasmid. Forced expression of the plasmid in CDCs would display the HHP/FLAG (red oval) on the surface of exosomes. **Figure S2.** Schematic illustration of mice treatment schedule. The TAC mice were randomly divided into 3 groups, PBS control, CON-EXO, and HHP-EXO (n = 12 each). Exosomes (4 mg/kg) or PBS were tail-vein injected on day 8, 10, 12, 14, 16, 18, 20 post-TAC. Echocardiographic studies were performed 3 days (Control) prior to, and on day 7, 14, 21, 28 and 42 after, the TAC. The mean arterial blood pressure was evaluated, serum was collected, and the hearts were harvested on day 42 post-TAC. **Figure S3.** Cardiac hypertrophy after exosome treatment. **A** Coronal sections of the hearts among groups by HE staining. **B** Quantitation of heart weight/body weight (left panel) and heart weight/tibial length (right panel) ratios among groups. Data are presented as ‘Mean ± STDEV’, n = 9-12 animals, *P < 0.05 and **P < 0.01. **Figure S4.** HHP-EXO and SC144 perform similar effect of inhibiting GP130-STAT pathway. H9C2 cardiomyocytes were pretreated with SC144 (10μM) for 1h, and then exposure to Ang II (1 μM) with or without HHP-EXO (50μg/ml) for 24h, the expression of β-MHC, GP130, p-STAT3, STAT3, p-ERK1/2, ERK, p-AKT and AKT was detected by Western blotting. **Table S1.** Parameters of cardiac function and related serum kinases levels in TAC mice with different treatments.** Table S2.** Reagents and antibodies used in the present study.** Table S3.** Primers for cloning of LAMP2b fusion plasmids used in the present study.

## Data Availability

The datasets used and/or analyzed during the current study are available from the corresponding author on reasonable request.

## Abbreviations

HHP: Heart homing peptide
CDCs: Cardiosphere-derived cells
TAC: Transverse aorta constriction
Ang II: Angiotensin II
CRIP2: Cysteine-rich protein 2
NRCMs: Neonatal rat cardiomyocytes
LVEF: Left ventricular ejection fraction
LVFS: Left ventricular fractional shortening
LVV,s: Left ventricular end-systolic volume
LVAW,d: Diastolic left ventricular anterior wall thickness
LVAW,s: Systolic left ventricular anterior wall thickness
LVH: Left ventricular hypertrophy
MBP: Mean blood pressure
NT-proBNP: N-terminal pro B-type natriuretic peptide
AMI: Acute myocardial infarction
GP130: Glycoprotein 130
