# Supplementary material for: Heart-targeting exosomes from human cardiosphere-derived cells improve the therapeutic effect on cardiac hypertrophy
Source: J Nanobiotechnology. 2022 Oct 4;20:435. doi: 10.1186/s12951-022-01630-3 (PMC9531502; doi:10.1186/s12951-022-01630-3)
Supplement: Supplementary file 1 — Additional file 1: Figure S1. Schematic illustration of displaying a homing peptide/FLAG on the surface of exosomes. The coding sequence of HHP/FLAG (red bar) was fused in-frame to the LAMP2b cDNA between the signal peptide (SP) and the N-terminus, which was then cloned into pLVX-IRES-ZsGreen1 expression plasmid. Forced expression of the plasmid in CDCs would display the HHP/FLAG (red oval) on the surface of exosomes. Figure S2. Schematic illustration of mice treatment schedule. The TAC mice were randomly divided into 3 groups, PBS control, CON-EXO, and HHP-EXO (n = 12 each). Exosomes (4 mg/kg) or PBS were tail-vein injected on day 8, 10, 12, 14, 16, 18, 20 post-TAC. Echocardiographic studies were performed 3 days (Control) prior to, and on day 7, 14, 21, 28 and 42 after, the TAC. The mean arterial blood pressure was evaluated, serum was collected, and the hearts were harvested on day 42 post-TAC. Figure S3. Cardiac hypertrophy after exosome treatment. A Coronal sections of the hearts among groups by HE staining. B Quantitation of heart weight/body weight (left panel) and heart weight/tibial length (right panel) ratios among groups. Data are presented as ‘Mean ± STDEV’, n = 9-12 animals, *P < 0.05 and **P < 0.01. Figure S4. HHP-EXO and SC144 perform similar effect of inhibiting GP130-STAT pathway. H9C2 cardiomyocytes were pretreated with SC144 (10μM) for 1h, and then exposure to Ang II (1 μM) with or without HHP-EXO (50μg/ml) for 24h, the expression of β-MHC, GP130, p-STAT3, STAT3, p-ERK1/2, ERK, p-AKT and AKT was detected by Western blotting. Table S1. Parameters of cardiac function and related serum kinases levels in TAC mice with different treatments. Table S2. Reagents and antibodies used in the present study. Table S3. Primers for cloning of LAMP2b fusion plasmids used in the present study. [file 12951_2022_1630_MOESM1_ESM.docx]

**Additional file**

**Heart-targeting exosomes from human cardiosphere-derived cells improve the therapeutic effect on cardiac hypertrophy**

Liang Mao^1,2^, Yun-Da Li^1^, Ruo-Lan Chen^1^, Gang Li^1^, Xiao-Xia Zhou^1^, Fei Song^1^, Chan Wu^1^, Yu Hu^1^, Yi-xiang Hong^1^, Xitong Dang^2^, Gui-Rong Li^1,3^, Yan Wang^1#^.

^1^ Xiamen Cardiovascular Hospital of Xiamen University, School of Medicine, Xiamen University, Xiamen 361000, China.

^2^ Key Laboratory of Medical Electrophysiology, Ministry of Education & Medical Electrophysiological Key Laboratory of Sichuan Province, (Collaborative Innovation Center for Prevention of Cardiovascular Diseases,) Institute of Cardiovascular Research, Southwest Medical University, Luzhou 646000, China.

^3^ Nanjing Amaigh Pharma Limited, Nanjing 210032, China.

**
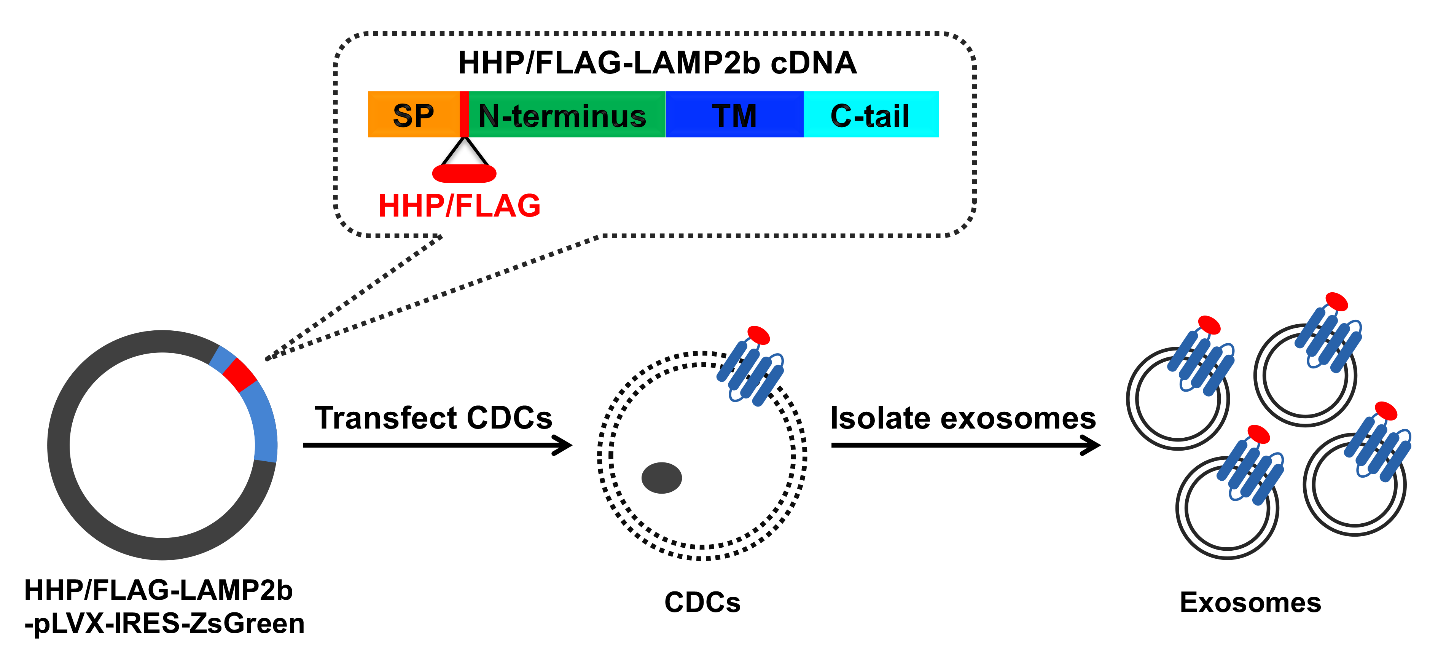
**

**Figure S1. Schematic illustration of displaying a homing peptide/FLAG on the surface of exosomes.** The coding sequence of HHP/FLAG (red bar) was fused in-frame to the LAMP2b cDNA between the signal peptide (SP) and the N-terminus, which was then cloned into pLVX-IRES-ZsGreen1 expression plasmid. Forced expression of the plasmid in CDCs would display the HHP/FLAG (red oval) on the surface of exosomes.

**
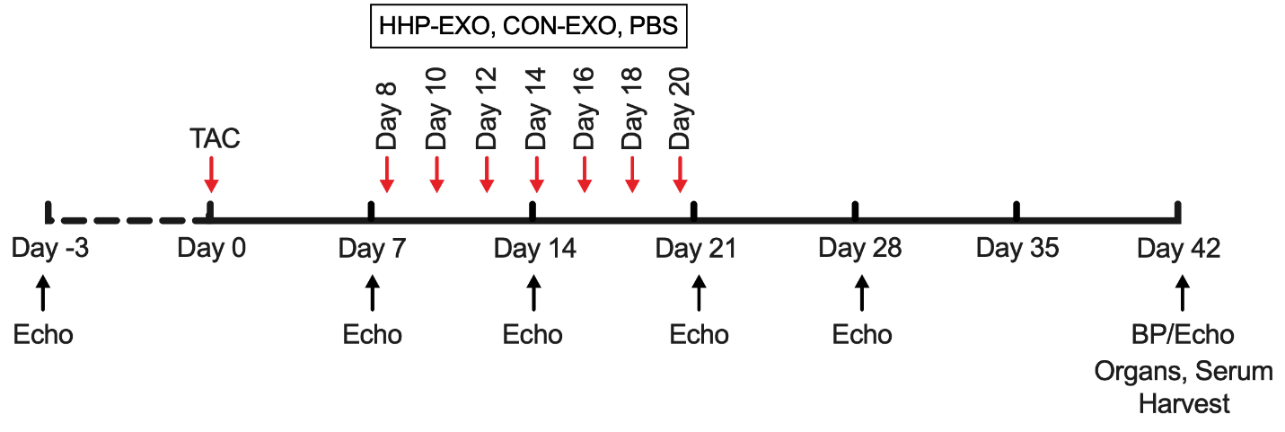
**

**Figure S2. Schematic illustration of mice treatment schedule**. The TAC mice were randomly divided into 3 groups, PBS control, CON-EXO, and HHP-EXO (n = 12 each). Exosomes (4 mg/kg) or PBS were tail-vein injected on day 8, 10, 12, 14, 16, 18, 20 post-TAC. Echocardiographic studies were performed 3 days (Control) prior to, and on day 7, 14, 21, 28 and 42 after, the TAC. The mean arterial blood pressure was evaluated, serum was collected, and the hearts were harvested on day 42 post-TAC.

**
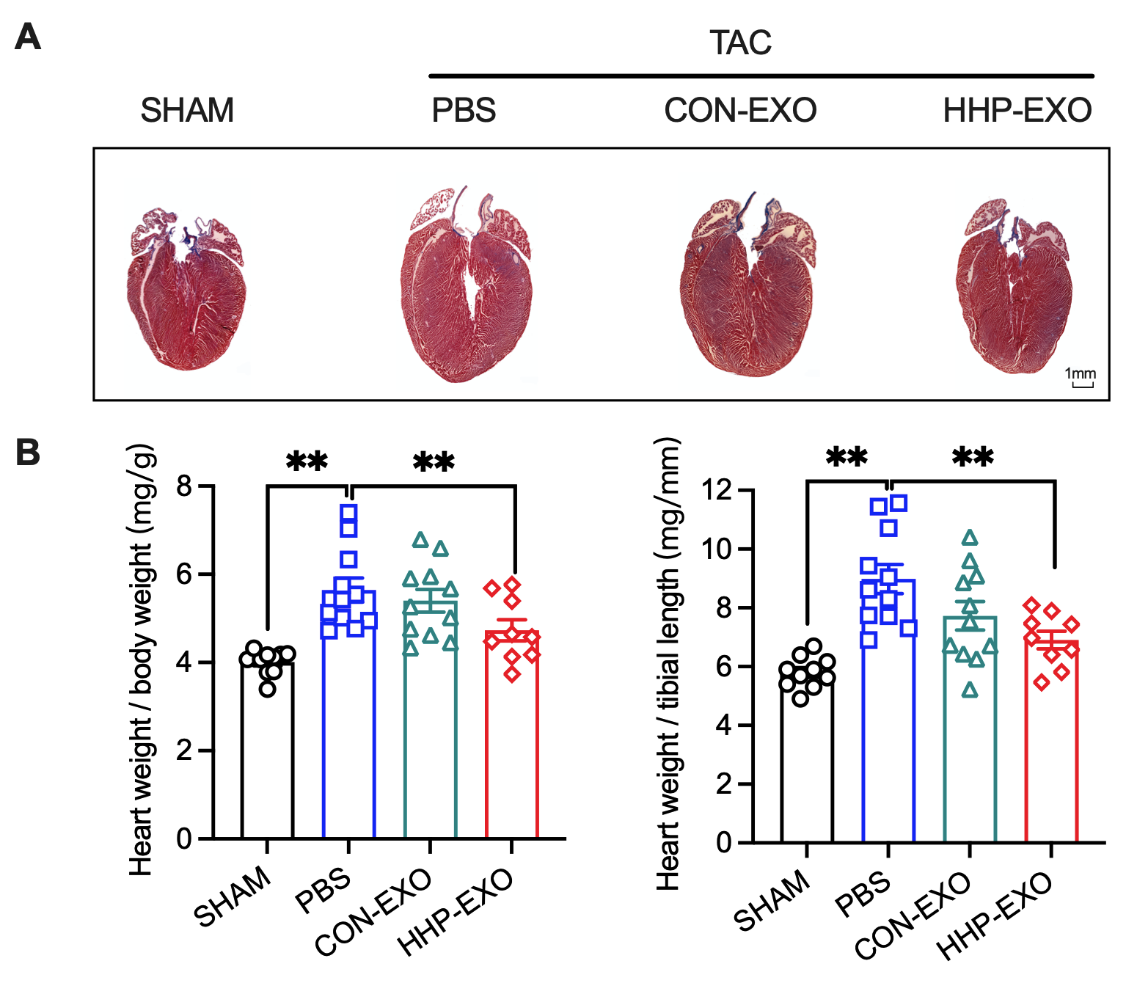
**

**Figure S3.** **Cardiac hypertrophy after exosome treatment.** **A** Coronal sections of the hearts among groups by HE staining. **B** Quantitation of heart weight/body weight (left panel) and heart weight/tibial length (right panel) ratios among groups. Data are presented as ‘Mean ± STDEV’, n = 9-12 animals, *P < 0.05 and **P < 0.01.


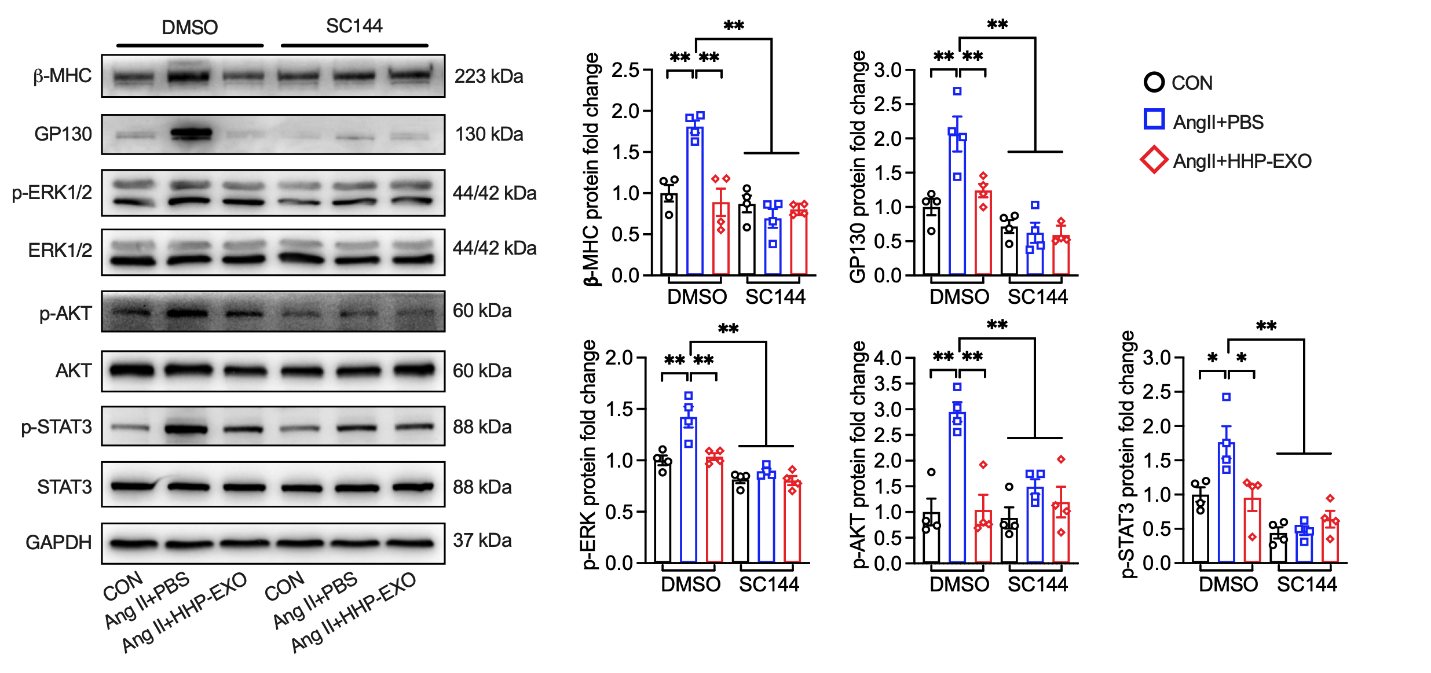


**Figure S4. HHP-EXO and SC144 perform similar effect of inhibiting GP130-STAT pathway.** H9C2 cardiomyocytes were pretreated with SC144 (10μM) for 1h, and then exposure to Ang II (1 μM) with or without HHP-EXO (50μg/ml) for 24h, the expression of β-MHC, GP130, p-STAT3, STAT3, p-ERK1/2, ERK, p-AKT and AKT was detected by Western blotting. Protein expression was normalized to that of GAPDH. Data are presented as Mean ± SEM, with *P < 0.05 and **P < 0.01.

**Table S1. Parameters of cardiac function and related serum kinases levels in TAC mice with different treatments**

|  | |  | TAC | | |
| --- | --- | --- | --- | --- | --- |
|  |  | SHAM  (n = 10) | PBS  (n = 12) | CON-EXO  (n = 12) | HHP-EXO  (n = 12) |
| LVEF | Control | 57.82% ± 0.97% | 55.25% ± 0.85% | 56.0% ± 2.15% | 55.45% ± 1.49% |
|  | Day 7 | 54.03% ± 1.86% | 44.73% ± 1.84%^**^ | 44.82% ± 1.46%^**^ | 46.37% ± 1.79%^**^ |
|  | Day 14 | 54.18% ± 0.88% | 44.37% ± 1.68%^**^ | 45.27% ± 1.52%^**^ | 44.10 %± 1.43%^**^ |
|  | Day 21 | 55.29% ± 1.15% | 39.45% ± 1.30%^**^ | 39.77% ± 1.30%^**^ | 44.40% ± 1.57%^**^ |
|  | Day 28 | 55.71% ± 1.54% | 38.94% ± 1.93%^**^ | 41.04% ± 1.80%^**^ | 46.85% ± 1.79%^**##^ |
|  | Day 42 | 53.27% ± 1.02% | 36.38% ± 1.75%^**^ | 36.24% ± 1.37%^**^ | 43.26% ± 2.16%^**##^ |
| LVFS | Control | 29.93% ± 0.63% | 28.26% ± 0.55% | 28.92% ± 1.43% | 28.50% ± 0.97% |
|  | Day 7 | 27.77% ± 1.34% | 22.02% ± 1.06%^**^ | 21.96% ± 0.85%^**^ | 23.02% ± 1.06%^**^ |
|  | Day 14 | 27.62% ± 0.56% | 21.82% ± 0.94%^**^ | 22.27% ± 0.88%^**^ | 21.49% ± 0.81%^**^ |
|  | Day 21 | 28.38% ± 0.74% | 18.96% ± 0.71%^**^ | 19.11% ± 0.71%^**^ | 21.74% ± 0.91%^**^ |
|  | Day 28 | 28.71% ± 1.01% | 18.52% ± 1.10%^**^ | 19.96% ± 1.05%^**^ | 23.57% ± 1.17%^**##^ |
|  | Day 42 | 27.00% ± 0.63% | 17.30% ± 0.94%^**^ | 17.24% ± 0.73%^**^ | 21.16% ± 1.24%^**##^ |
| BNP (pg/ml) | | 32.75 ± 3.38 | 51.86 ± 2.33^**^ | 38.11 ± 4.27 | 32.10 ± 4.67^##^ |
| MAP (mmHg) | | 89.65 ± 3.00 | 116.90 ± 4.76^**^ | 103.40 ± 3.06 | 94.38 ± 4.87^##^ |
| Ang II (pg/ml) | | 196.10 ± 17.93 | 280.90 ± 12.53^**^ | 223.40 ± 18.53^##^ | 190.60 ± 13.00^##^ |
| Creatinine (ug/ml) | | 4.13 ± 0.17 | 4.89 ± 0.24 | 5.28 ± 0.41 | 4.59 ± 0.30 |
| Lung wet/dry (g/g) | | 4.34 ± 0.03 | 4.51 ± 0.04^**^ | 4.47 ± 0.04 | 4.29 ± 0.04^##^ |
| Kidney/body (mg/g) | | 1.22 ± 0.03 | 1.17 ± 0.03 | 1.08 ± 0.02 | 1.15 ± 0.03 |
| HW/BW (mg/g) | | 0.40 ± 0.01 | 0.60 ± 0.05^**^ | 0.54 ± 0.03 | 0.47 ± 0.02^##^ |
| HW/TL (mg/mm) | | 0.58 ± 0.02 | 0.88 ± 0.06^**^ | 0.77 ± 0.05 | 0.69 ± 0.03^##^ |

Compared with Sham * p < 0.05, ** p < 0.01, compared with PBS # p < 0.05, ## p < 0.01.

**Table S2. Reagents and antibodies used in the present study**

| **Name** | **Vendor or Source** | **Catalog #** |
| --- | --- | --- |
| Iscove’s modified Dulbecco’s medium (IMDM) | Thermo Fisher Scientific (MA, USA) | 12440046 |
| Fetal bovine serum (FBS) | Thermo Fisher Scientific (MA, USA) | 10099141 |
| Dulbecco's modified Eagle medium (DMEM) | Thermo Fisher Scientific (MA, USA) | 11995040 |
| Penicillin streptomycin solution | Thermo Fisher Scientific (MA, USA) | 15140122 |
| Trizol | Thermo Fisher Scientific (MA, USA) | 15596026 |
| RevertAid first strand cDNA synthesis kit | Thermo Fisher Scientific (MA, USA) | K1622 |
| CellTracker™ CM-DiI Dye | Thermo Fisher Scientific (MA, USA) | C7000 |
| FITC conjugated wheat germ agglutinin (FITC-WGA) | Merck Millipore (Darmstadt, Germany) | L4895 |
| FITC-phalloidin | Merck Millipore (Darmstadt, Germany) | P5282 |
| 4’6-diamidino-2-phenylindole (DAPI) | Merck Millipore (Darmstadt, Germany) | P9542 |
| Angiotensin II | Merck Millipore (Darmstadt, Germany) | 05-23-0101 |
| ANTI-FLAG^®^ M2 magnetic bead | Merck Millipore (Darmstadt, Germany) | M8823 |
| Immobilon western chemiluminescent HRP substrate | Merck Millipore (Darmstadt, Germany) | WBKLS0500 |
| QuantiNova SYBR Green PCR kit | QIAGEN (Hilden, Germany) | 208052 |
| The hematoxylin-eosin staining kit | Solarbio Life Sciences (Beijing, China) | G1120 |
| Masson’s staining kit | Solarbio Life Sciences (Beijing, China) | G1371 |
| Mouse angiotensin Ⅱ ELISA kit | Elabscience Biotechnology  (Wuhan, China) | E-EL-M2612c |
| Mouse N-terminal pro-brain  natriuretic peptide ELISA kit | Elabscience Biotechnology  (Wuhan, China) | E-EL-M0834c |
| Creatinine (Cr) colorimetric  assay kit | Elabscience Biotechnology  (Wuhan, China) | E-BC-K188-M |
| Anti-beta-MHC | Abcam Biotechnology  (Cambridge, MA, USA). | ab50967 |
| anti-BNP | Abcam Biotechnology  (Cambridge, MA, USA). | ab239510 |
| anti-STAT3 | Abcam Biotechnology  (Cambridge, MA, USA). | ab68153 |
| anti-phospho-STAT3  (p-Tyr705) | Abcam Biotechnology  (Cambridge, MA, USA). | ab76315 |
| anti-GAPDH | Abcam Biotechnology  (Cambridge, MA, USA). | ab181602 |
| anti-Tubulin | Abcam Biotechnology  (Cambridge, MA, USA). | ab44928 |
| Anti-gp130 | Cell Signaling Technology  (Danvers, MA, USA). | #3732 |
| anti-AKT | Cell Signaling Technology  (Danvers, MA, USA). | #4691 |
| anti-phospho-AKT (Ser473) | Cell Signaling Technology  (Danvers, MA, USA). | #4060 |
| anti-p44/42 MAPK (ERK1/2) | Cell Signaling Technology  (Danvers, MA, USA). | #9102 |
| anti-phospho-p44/42 MAPK  (ERK1/2) (Thr202/Tyr204) | Cell Signaling Technology  (Danvers, MA, USA). | #4370 |
| Anti-β-actin | Santa Cruz (Dallas, TX, USA). | sc-47778 |
| anti-CD9 | Santa Cruz (Dallas, TX, USA). | sc-13118 |
| anti-ALIX | Santa Cruz (Dallas, TX, USA). | sc-53540 |
| anti-TSG101 | Santa Cruz (Dallas, TX, USA). | sc-7964 |
| Peroxidase AffiniPure Goat  Anti-Mouse IgG （H+L） | Jackson ImmunoResearch  (West Grove, PA, USA). | 115-035-003 |
| Peroxidase-AffiniPure Goat  Anti-Rabbit IgG (H+L) | Jackson ImmunoResearch  (West Grove, PA, USA). | 111-035-003 |

**Table S3. Primers for cloning of LAMP2b fusion plasmids used in the present study**

| Name | DNA sequence (5′-3′) |
| --- | --- |
| LAMP-F | 5′-TATGCTCGAGTGCGGGGTCATGGTGTGCT-3′ |
| LAMP-R | 5′-ATCTGGATCCTTACAGAGTCTGATATCCAG-3′ |
| HHP-F | 5′-TGCCGACCTCCGCGTCGCCGACGGCGTCGACGCCGGCGACGCTCA  GAAAATGCCACTTGCCT-3 |
| HHP-R | 5′-GCGTCGCCGGCGTCGACGCCGTCGGCGACGCGGAGGTCGGCAATC  TGTCAAATTAAGTTCCA-3′ |
| FLAG-F | 5′- GATTACAAGGATGACGATGACAAGTCAGAAAATGCCACTTGCCT-3′ |
| FLAG-R | 5′- CTTGTCATCGTCATCCTTGTAATCATCTGTCAAATTAAGTTCCA-3′ |
